# Ethyl 1-(2,4-dichloro­benz­yl)-4-oxo-7-trifluoro­meth­yl-1,4-dihydro­quinoline-3-carboxyl­ate

**DOI:** 10.1107/S1600536812001249

**Published:** 2012-01-18

**Authors:** Hoong-Kun Fun, Chin Wei Ooi, B. Garudachari, Arun M. Isloor, Gurumurthy Hegde

**Affiliations:** a X-ray Crystallography Unit, School of Physics, Universiti Sains Malaysia, 11800 USM, Penang, Malaysia; b Department of Chemistry, National Institute of Technology-Karnataka, Surathkal, Mangalore 575 025, India; c Faculty of Industrial Science and Technology, Universiti Malaysia Pahang, Lebuhraya Tun Razak, 26300 Gambang Kuantan, Pahang Darul Makmur, Malaysia

## Abstract

In the title compound, C_20_H_14_Cl_2_F_3_NO_3_, the trifluromethyl group is disordered over two sets of sites in a 0.784 (10):0.216 (10) ratio. The quinoline ring system is essentially planar with a maximum deviation of 0.058 (2) Å for the N atom and forms dihedral angles of 89.23 (11) and 8.13 (17)°, respectively with the mean planes of the benzene ring and the carboxyl­ate group. In the crystal, pairs of weak C—H⋯O and C—H⋯F hydrogen bonds link mol­ecules into centrosymmetric dimers. The crystal structure is further stabilized by weak π–π [centroid–centroid distance = 3.624 (2) Å] inter­actions.

## Related literature

For background to the properties and uses of quinoline derivatives, see: Kaur *et al.* (2010 ▶); Eswaran *et al.* (2010 ▶); Chou *et al.* (2010 ▶); Chen *et al.* (2004 ▶); Shingalapur *et al.* (2009 ▶). For a related structure, see: Fun *et al.* (2011 ▶). For standard bond-length data, see: Allen *et al.* (1987 ▶).
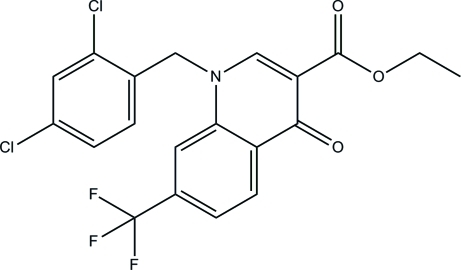


## Experimental

### 

#### Crystal data

C_20_H_14_Cl_2_F_3_NO_3_
*M* *_r_* = 444.22
Triclinic, 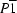

*a* = 8.090 (2) Å
*b* = 9.547 (3) Å
*c* = 14.047 (4) Å
α = 77.299 (6)°
β = 76.198 (5)°
γ = 67.488 (4)°
*V* = 963.3 (5) Å^3^
*Z* = 2
Mo *K*α radiation
μ = 0.39 mm^−1^
*T* = 296 K
0.43 × 0.18 × 0.07 mm

#### Data collection

Bruker APEX DUO CCD area-detector diffractometer
Absorption correction: multi-scan (*SADABS*; Bruker, 2009 ▶) *T*_min_ = 0.852, *T*_max_ = 0.972
13916 measured reflections
5071 independent reflections
2759 reflections with *I* > 2σ(*I*)
*R*_int_ = 0.027

#### Refinement

*R*[*F* ^2^ > 2σ(*F* ^2^)] = 0.056
*wR*(*F* ^2^) = 0.179
*S* = 1.04
5071 reflections
288 parameters
7 restraints
H-atom parameters constrained
Δρ_max_ = 0.35 e Å^−3^
Δρ_min_ = −0.34 e Å^−3^

### 

Data collection: *APEX2* (Bruker, 2009 ▶); cell refinement: *SAINT* (Bruker, 2009 ▶); data reduction: *SAINT*; program(s) used to solve structure: *SHELXTL* (Sheldrick, 2008 ▶); program(s) used to refine structure: *SHELXTL*; molecular graphics: *SHELXTL*; software used to prepare material for publication: *SHELXTL* and *PLATON* (Spek, 2009 ▶).

## Supplementary Material

Crystal structure: contains datablock(s) global, I. DOI: 10.1107/S1600536812001249/lh5400sup1.cif

Structure factors: contains datablock(s) I. DOI: 10.1107/S1600536812001249/lh5400Isup2.hkl

Supplementary material file. DOI: 10.1107/S1600536812001249/lh5400Isup3.cml

Additional supplementary materials:  crystallographic information; 3D view; checkCIF report

## Figures and Tables

**Table 1:** Hydrogen-bond geometry (Å, °)

*D*—H⋯*A*	*D*—H	H⋯*A*	*D*⋯*A*	*D*—H⋯*A*
C16—H16*A*⋯O3^i^	0.93	2.52	3.292 (5)	141
C18—H18*A*⋯F2^i^	0.97	2.50	3.355 (7)	147

Symmetry code: (i) 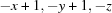.

**Fig. 1.**
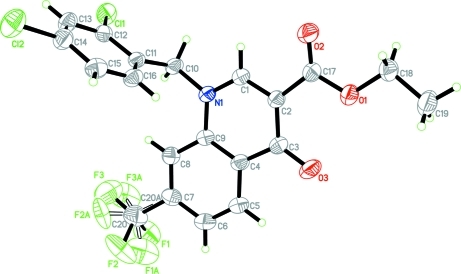
The molecular structure of the title compound, showing 30% probability displacement ellipsoids. Both disorder components are shown.

**Fig. 2.**
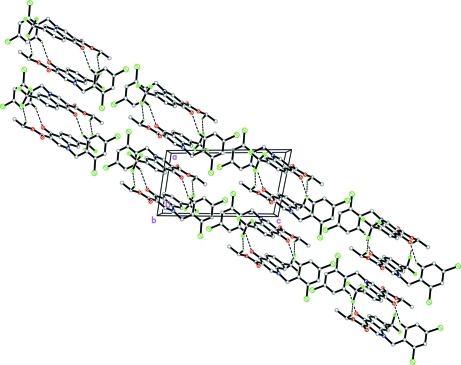
The crystal packing of the title compound. The dashed lines represent the hydrogen bonds. Only the major disordered component is shown.

## supplementary crystallographic information

### Comment

The quinoline moiety is of great importance to chemists as well as biologists as it is one of the key building elements for many naturally occurring compounds. Members of this family have wide range of applications as pharmaceuticals as antimalarial (Kaur *et al.*, 2010), anti-tuberculosis (Eswaran *et al.*, 2010), antitumor (Chou *et al.*, 2010), anticancer (Chen *et al.*, 2004) and antiviral (Shingalapur *et al.*, 2009) agents. Some of the present day drugs such as chloroquine, mefloquine, tafenoquine and primaquine contain quinoline as the basic unit in their structures. In view of the biological importance, we have synthesized the title compound to study its crystal structure.

In the molecular structure (Fig. 1), the trifluromethyl group is disordered over two sets of sites in a ratio of 0.784 (10):0.216 (10). The quinoline ring (N1/C1–C9) is essentially planar with a maximum deviation of 0.058 (2) Å at atom N1. The quinoline ring makes dihedral angles of 89.23 (11) and 8.13 (17)°, respectively with the chloro-substituted benzene ring (C11–C16) and the carboxylate group (O1/O2/C17–C19). The bond lengths (Allen *et al.*, 1987) and angles are within normal ranges and are comparable to a related structure (Fun *et al.*, 2011).

In the crystal (Fig. 2), intermolecular C16—H16A···O3^i^ and C18—H18A···F2^i^ hydrogen bonds (Table 1) link molecules to form dimers. The crystal is further stabilized by weak π–π interactions between the quinoline (N1/C1–C9) and the benzene ring (C4–C9) [centroid-to-centroid (-*x*, 1 - *y*, -*z*); distance = 3.624 (2) Å].

### Experimental

A mixture of ethyl 4-hydroxy-7-(trifluoromethyl)quinoline-3-carboxylate (0.10 g, 0.00035 mol), potassium carbonate (0.053 g, 0.00038 mol) and 1-(bromomethyl)-2,4-dichlorobenzene (0.091 g, 0.00038 mol) in dimethylformamide (5 ml) was stirred at 353K for 3 h. After completion of the reaction, the reaction mixture was poured into ice-cold water. The solid product obtained was filtered, washed with water and recrystallized using ethanol. Yield: 0.150 g, 96.77%. *M. p.*: 428–429 K.

### Refinement

All H atoms were positioned geometrically and refined using a riding model with *U*_iso_(H) = 1.2 or 1.5*U*_eq_(C) (C—H = 0.93, 0.96 or 0.97 Å). A rotating group model was applied to the methyl group. In the final refinement, the outliners (-4 - 6 1), (3 2 0), (5 0 6), (5 1 8) were omitted. A bond-distance restraint was applied to C20A–C7. The same *U*_ij_ parameters were used for atom pairs F3A/F1A and C20A/C7.

### Crystal data

**Table:**

C_20_H_14_Cl_2_F_3_NO_3_	*Z* = 2
*M**_r_* = 444.22	*F*(000) = 452
Triclinic, *P*1	*D*_x_ = 1.531 Mg m^−^^3^
Hall symbol: -P 1	Mo *K*α radiation, λ = 0.71073 Å
*a* = 8.090 (2) Å	Cell parameters from 3077 reflections
*b* = 9.547 (3) Å	θ = 2.3–24.2°
*c* = 14.047 (4) Å	µ = 0.39 mm^−^^1^
α = 77.299 (6)°	*T* = 296 K
β = 76.198 (5)°	Block, colourless
γ = 67.488 (4)°	0.43 × 0.18 × 0.07 mm
*V* = 963.3 (5) Å^3^	

### Data collection

**Table:**

Bruker APEX DUO CCD area-detector diffractometer	5071 independent reflections
Radiation source: fine-focus sealed tube	2759 reflections with *I* > 2σ(*I*)
graphite	*R*_int_ = 0.027
φ and ω scans	θ_max_ = 29.1°, θ_min_ = 1.5°
Absorption correction: multi-scan (*SADABS*; Bruker, 2009)	*h* = −10→11
*T*_min_ = 0.852, *T*_max_ = 0.972	*k* = −13→13
13916 measured reflections	*l* = −19→19

### Refinement

**Table:**

Refinement on *F*^2^	Primary atom site location: structure-invariant direct methods
Least-squares matrix: full	Secondary atom site location: difference Fourier map
*R*[*F*^2^ > 2σ(*F*^2^)] = 0.056	Hydrogen site location: inferred from neighbouring sites
*wR*(*F*^2^) = 0.179	H-atom parameters constrained
*S* = 1.03	*w* = 1/[σ^2^(*F*_o_^2^) + (0.0711*P*)^2^ + 0.3522*P*] where *P* = (*F*_o_^2^ + 2*F*_c_^2^)/3
5071 reflections	(Δ/σ)_max_ < 0.001
288 parameters	Δρ_max_ = 0.35 e Å^−^^3^
7 restraints	Δρ_min_ = −0.34 e Å^−^^3^

### Special details

**Table:**

Geometry. All e.s.d.'s (except the e.s.d. in the dihedral angle between two l.s. planes) are estimated using the full covariance matrix. The cell e.s.d.'s are taken into account individually in the estimation of e.s.d.'s in distances, angles and torsion angles; correlations between e.s.d.'s in cell parameters are only used when they are defined by crystal symmetry. An approximate (isotropic) treatment of cell e.s.d.'s is used for estimating e.s.d.'s involving l.s. planes.
Refinement. Refinement of *F*^2^ against ALL reflections. The weighted *R*-factor *wR* and goodness of fit *S* are based on *F*^2^, conventional *R*-factors *R* are based on *F*, with *F* set to zero for negative *F*^2^. The threshold expression of *F*^2^ > σ(*F*^2^) is used only for calculating *R*-factors(gt) *etc*. and is not relevant to the choice of reflections for refinement. *R*-factors based on *F*^2^ are statistically about twice as large as those based on *F*, and *R*- factors based on ALL data will be even larger.

### Fractional atomic coordinates and isotropic or equivalent isotropic displacement parameters (Å^2^)

**Table:**

	*x*	*y*	*z*	*U*_iso_*/*U*_eq_	Occ. (<1)
Cl1	−0.33323 (10)	0.73631 (10)	0.39029 (6)	0.0780 (3)	
Cl2	0.24014 (16)	0.66624 (13)	0.54781 (7)	0.1052 (4)	
C20	0.1448 (11)	0.0460 (8)	0.2381 (6)	0.0900 (15)	0.784 (10)
F1	0.0710 (8)	−0.0345 (4)	0.2048 (3)	0.1144 (17)	0.784 (10)
F2	0.2991 (6)	−0.0610 (5)	0.2577 (5)	0.137 (2)	0.784 (10)
F3	0.0375 (13)	0.0915 (6)	0.3154 (4)	0.197 (5)	0.784 (10)
C20A	0.134 (3)	0.055 (2)	0.2391 (13)	0.0638 (7)	0.216 (10)
F1A	0.207 (3)	−0.0777 (17)	0.2083 (14)	0.149 (7)	0.216 (10)
F2A	0.204 (2)	0.042 (2)	0.3172 (9)	0.103 (6)	0.216 (10)
F3A	−0.026 (2)	0.078 (2)	0.2781 (19)	0.149 (7)	0.216 (10)
O1	0.3593 (3)	0.7747 (2)	−0.19081 (13)	0.0747 (6)	
O2	0.2188 (3)	0.9285 (2)	−0.07639 (15)	0.0818 (6)	
O3	0.4032 (4)	0.4757 (3)	−0.14202 (17)	0.0992 (8)	
N1	0.0638 (3)	0.5929 (2)	0.11707 (14)	0.0525 (5)	
C1	0.1139 (3)	0.6989 (3)	0.04830 (18)	0.0538 (6)	
H1A	0.0652	0.7997	0.0611	0.065*	
C2	0.2309 (3)	0.6694 (3)	−0.03871 (18)	0.0519 (6)	
C3	0.3065 (4)	0.5147 (3)	−0.0630 (2)	0.0614 (7)	
C4	0.2558 (3)	0.4008 (3)	0.01455 (19)	0.0546 (6)	
C5	0.3257 (4)	0.2471 (3)	−0.0008 (2)	0.0668 (7)	
H5A	0.4018	0.2206	−0.0600	0.080*	
C6	0.2850 (4)	0.1353 (3)	0.0693 (2)	0.0726 (8)	
H6A	0.3294	0.0344	0.0570	0.087*	
C7	0.1763 (4)	0.1747 (3)	0.1588 (2)	0.0638 (7)	
C8	0.1019 (4)	0.3237 (3)	0.1768 (2)	0.0580 (6)	
H8A	0.0275	0.3481	0.2369	0.070*	
C9	0.1394 (3)	0.4388 (3)	0.10383 (18)	0.0506 (6)	
C10	−0.0705 (3)	0.6443 (3)	0.20509 (18)	0.0570 (6)	
H10A	−0.1461	0.5812	0.2247	0.068*	
H10B	−0.1484	0.7490	0.1876	0.068*	
C11	0.0122 (3)	0.6370 (3)	0.29170 (17)	0.0497 (6)	
C12	−0.0999 (3)	0.6848 (3)	0.37876 (18)	0.0546 (6)	
C13	−0.0312 (4)	0.6930 (3)	0.4573 (2)	0.0692 (8)	
H13A	−0.1089	0.7262	0.5148	0.083*	
C14	0.1529 (4)	0.6517 (3)	0.4499 (2)	0.0680 (8)	
C15	0.2693 (4)	0.5999 (3)	0.3659 (2)	0.0651 (7)	
H15A	0.3942	0.5695	0.3619	0.078*	
C16	0.1983 (4)	0.5938 (3)	0.2878 (2)	0.0580 (6)	
H16A	0.2769	0.5598	0.2307	0.070*	
C17	0.2670 (3)	0.8046 (3)	−0.10207 (19)	0.0566 (6)	
C18	0.3971 (5)	0.9011 (3)	−0.2582 (2)	0.0729 (8)	
H18A	0.4611	0.9439	−0.2290	0.087*	
H18B	0.2848	0.9811	−0.2730	0.087*	
C19	0.5111 (6)	0.8397 (4)	−0.3499 (2)	0.1007 (13)	
H19A	0.5278	0.9226	−0.3993	0.151*	
H19B	0.4521	0.7878	−0.3740	0.151*	
H19C	0.6271	0.7691	−0.3356	0.151*	

### Atomic displacement parameters (Å^2^)

**Table:**

	*U*^11^	*U*^22^	*U*^33^	*U*^12^	*U*^13^	*U*^23^
Cl1	0.0572 (4)	0.0910 (6)	0.0667 (5)	−0.0187 (4)	0.0125 (3)	−0.0118 (4)
Cl2	0.1179 (8)	0.1260 (8)	0.0906 (7)	−0.0464 (7)	−0.0256 (6)	−0.0378 (6)
C20	0.091 (4)	0.062 (3)	0.116 (4)	−0.028 (3)	−0.016 (3)	−0.010 (3)
F1	0.120 (4)	0.097 (2)	0.156 (3)	−0.073 (3)	−0.038 (2)	0.003 (2)
F2	0.099 (3)	0.099 (3)	0.207 (5)	−0.048 (2)	−0.072 (3)	0.069 (3)
F3	0.325 (11)	0.088 (3)	0.129 (4)	−0.094 (5)	0.092 (6)	−0.020 (3)
C20A	0.0626 (17)	0.0525 (15)	0.0778 (19)	−0.0208 (13)	−0.0151 (14)	−0.0079 (13)
F1A	0.096 (9)	0.116 (10)	0.226 (15)	−0.066 (7)	0.006 (8)	0.010 (9)
F2A	0.083 (9)	0.145 (15)	0.076 (8)	−0.057 (10)	−0.038 (7)	0.053 (8)
F3A	0.096 (9)	0.116 (10)	0.226 (15)	−0.066 (7)	0.006 (8)	0.010 (9)
O1	0.0975 (15)	0.0655 (12)	0.0540 (11)	−0.0334 (11)	0.0120 (10)	−0.0122 (9)
O2	0.1094 (17)	0.0645 (13)	0.0696 (13)	−0.0377 (12)	0.0106 (12)	−0.0200 (10)
O3	0.1143 (19)	0.0721 (14)	0.0803 (15)	−0.0225 (13)	0.0394 (13)	−0.0285 (12)
N1	0.0536 (12)	0.0498 (11)	0.0481 (11)	−0.0138 (9)	0.0006 (9)	−0.0124 (9)
C1	0.0558 (15)	0.0509 (14)	0.0526 (14)	−0.0154 (11)	−0.0049 (11)	−0.0139 (11)
C2	0.0520 (14)	0.0529 (14)	0.0486 (13)	−0.0178 (11)	−0.0027 (11)	−0.0103 (11)
C3	0.0539 (15)	0.0601 (16)	0.0569 (15)	−0.0089 (12)	0.0025 (12)	−0.0153 (12)
C4	0.0477 (13)	0.0523 (14)	0.0582 (15)	−0.0091 (11)	−0.0061 (11)	−0.0153 (11)
C5	0.0615 (17)	0.0576 (16)	0.0692 (18)	−0.0067 (13)	−0.0025 (13)	−0.0198 (14)
C6	0.0751 (19)	0.0515 (16)	0.087 (2)	−0.0132 (14)	−0.0137 (16)	−0.0176 (15)
C7	0.0626 (17)	0.0525 (15)	0.0778 (19)	−0.0208 (13)	−0.0151 (14)	−0.0079 (13)
C8	0.0548 (15)	0.0585 (16)	0.0601 (15)	−0.0212 (12)	−0.0063 (12)	−0.0087 (12)
C9	0.0476 (13)	0.0481 (13)	0.0553 (14)	−0.0139 (11)	−0.0070 (11)	−0.0131 (11)
C10	0.0511 (14)	0.0554 (14)	0.0551 (15)	−0.0129 (11)	0.0065 (11)	−0.0168 (12)
C11	0.0544 (14)	0.0411 (12)	0.0491 (13)	−0.0184 (10)	0.0034 (10)	−0.0076 (10)
C12	0.0558 (14)	0.0498 (14)	0.0491 (14)	−0.0196 (11)	0.0069 (11)	−0.0039 (11)
C13	0.078 (2)	0.0700 (18)	0.0518 (16)	−0.0258 (15)	0.0095 (14)	−0.0156 (13)
C14	0.081 (2)	0.0682 (18)	0.0591 (16)	−0.0310 (16)	−0.0091 (15)	−0.0132 (14)
C15	0.0621 (17)	0.0616 (17)	0.0757 (19)	−0.0261 (14)	−0.0078 (14)	−0.0140 (14)
C16	0.0562 (15)	0.0569 (15)	0.0578 (15)	−0.0208 (12)	0.0045 (12)	−0.0156 (12)
C17	0.0541 (15)	0.0618 (16)	0.0529 (14)	−0.0210 (12)	−0.0026 (11)	−0.0119 (12)
C18	0.084 (2)	0.0724 (19)	0.0620 (17)	−0.0388 (16)	0.0061 (15)	−0.0073 (14)
C19	0.137 (3)	0.100 (3)	0.064 (2)	−0.058 (3)	0.023 (2)	−0.0218 (18)

### Geometric parameters (Å, °)

**Table:**

Cl1—C12	1.735 (3)	C5—H5A	0.9300
Cl2—C14	1.741 (3)	C6—C7	1.388 (4)
C20—F3	1.262 (8)	C6—H6A	0.9300
C20—F2	1.320 (7)	C7—C8	1.371 (4)
C20—F1	1.344 (8)	C8—C9	1.401 (4)
C20—C7	1.522 (8)	C8—H8A	0.9300
C20A—F3A	1.235 (16)	C10—C11	1.499 (4)
C20A—F1A	1.303 (16)	C10—H10A	0.9700
C20A—F2A	1.311 (16)	C10—H10B	0.9700
C20A—C7	1.494 (17)	C11—C12	1.387 (3)
O1—C17	1.320 (3)	C11—C16	1.391 (4)
O1—C18	1.445 (3)	C12—C13	1.377 (4)
O2—C17	1.204 (3)	C13—C14	1.371 (4)
O3—C3	1.236 (3)	C13—H13A	0.9300
N1—C1	1.349 (3)	C14—C15	1.376 (4)
N1—C9	1.396 (3)	C15—C16	1.375 (4)
N1—C10	1.472 (3)	C15—H15A	0.9300
C1—C2	1.365 (3)	C16—H16A	0.9300
C1—H1A	0.9300	C18—C19	1.485 (4)
C2—C3	1.450 (4)	C18—H18A	0.9700
C2—C17	1.484 (4)	C18—H18B	0.9700
C3—C4	1.465 (4)	C19—H19A	0.9600
C4—C5	1.401 (4)	C19—H19B	0.9600
C4—C9	1.402 (3)	C19—H19C	0.9600
C5—C6	1.367 (4)		
			
F3—C20—F2	112.3 (8)	N1—C9—C8	121.8 (2)
F3—C20—F1	105.5 (6)	N1—C9—C4	118.2 (2)
F2—C20—F1	101.2 (6)	C8—C9—C4	120.1 (2)
F3—C20—C7	113.9 (6)	N1—C10—C11	113.9 (2)
F2—C20—C7	111.8 (6)	N1—C10—H10A	108.8
F1—C20—C7	111.2 (6)	C11—C10—H10A	108.8
F3A—C20A—F1A	110.4 (17)	N1—C10—H10B	108.8
F3A—C20A—F2A	99.9 (16)	C11—C10—H10B	108.8
F1A—C20A—F2A	106.3 (18)	H10A—C10—H10B	107.7
F3A—C20A—C7	118.3 (17)	C12—C11—C16	117.1 (2)
F1A—C20A—C7	109.6 (14)	C12—C11—C10	119.6 (2)
F2A—C20A—C7	111.4 (13)	C16—C11—C10	123.2 (2)
C17—O1—C18	116.3 (2)	C13—C12—C11	121.8 (3)
C1—N1—C9	120.0 (2)	C13—C12—Cl1	118.4 (2)
C1—N1—C10	118.3 (2)	C11—C12—Cl1	119.8 (2)
C9—N1—C10	121.7 (2)	C14—C13—C12	119.3 (3)
N1—C1—C2	124.9 (2)	C14—C13—H13A	120.3
N1—C1—H1A	117.6	C12—C13—H13A	120.3
C2—C1—H1A	117.6	C13—C14—C15	120.8 (3)
C1—C2—C3	119.4 (2)	C13—C14—Cl2	119.5 (2)
C1—C2—C17	115.1 (2)	C15—C14—Cl2	119.7 (3)
C3—C2—C17	125.5 (2)	C16—C15—C14	119.1 (3)
O3—C3—C2	125.5 (3)	C16—C15—H15A	120.4
O3—C3—C4	120.0 (2)	C14—C15—H15A	120.4
C2—C3—C4	114.5 (2)	C15—C16—C11	121.8 (2)
C5—C4—C9	118.3 (2)	C15—C16—H16A	119.1
C5—C4—C3	118.9 (2)	C11—C16—H16A	119.1
C9—C4—C3	122.8 (2)	O2—C17—O1	122.9 (2)
C6—C5—C4	121.6 (3)	O2—C17—C2	124.4 (2)
C6—C5—H5A	119.2	O1—C17—C2	112.6 (2)
C4—C5—H5A	119.2	O1—C18—C19	107.1 (3)
C5—C6—C7	119.0 (3)	O1—C18—H18A	110.3
C5—C6—H6A	120.5	C19—C18—H18A	110.3
C7—C6—H6A	120.5	O1—C18—H18B	110.3
C8—C7—C6	121.6 (3)	C19—C18—H18B	110.3
C8—C7—C20A	117.5 (8)	H18A—C18—H18B	108.6
C6—C7—C20A	120.9 (8)	C18—C19—H19A	109.5
C8—C7—C20	120.7 (4)	C18—C19—H19B	109.5
C6—C7—C20	117.7 (4)	H19A—C19—H19B	109.5
C7—C8—C9	119.3 (3)	C18—C19—H19C	109.5
C7—C8—H8A	120.4	H19A—C19—H19C	109.5
C9—C8—H8A	120.4	H19B—C19—H19C	109.5
			
C9—N1—C1—C2	3.1 (4)	C20A—C7—C8—C9	179.4 (8)
C10—N1—C1—C2	−177.2 (2)	C20—C7—C8—C9	177.5 (4)
N1—C1—C2—C3	1.6 (4)	C1—N1—C9—C8	174.9 (2)
N1—C1—C2—C17	−179.3 (2)	C10—N1—C9—C8	−4.8 (4)
C1—C2—C3—O3	174.7 (3)	C1—N1—C9—C4	−5.0 (4)
C17—C2—C3—O3	−4.3 (5)	C10—N1—C9—C4	175.2 (2)
C1—C2—C3—C4	−3.8 (4)	C7—C8—C9—N1	178.3 (2)
C17—C2—C3—C4	177.2 (2)	C7—C8—C9—C4	−1.7 (4)
O3—C3—C4—C5	3.0 (4)	C5—C4—C9—N1	−177.3 (2)
C2—C3—C4—C5	−178.4 (3)	C3—C4—C9—N1	2.6 (4)
O3—C3—C4—C9	−176.8 (3)	C5—C4—C9—C8	2.7 (4)
C2—C3—C4—C9	1.8 (4)	C3—C4—C9—C8	−177.4 (2)
C9—C4—C5—C6	−0.8 (4)	C1—N1—C10—C11	−93.1 (3)
C3—C4—C5—C6	179.3 (3)	C9—N1—C10—C11	86.7 (3)
C4—C5—C6—C7	−2.1 (5)	N1—C10—C11—C12	178.9 (2)
C5—C6—C7—C8	3.2 (5)	N1—C10—C11—C16	3.4 (3)
C5—C6—C7—C20A	−177.6 (8)	C16—C11—C12—C13	1.6 (4)
C5—C6—C7—C20	−175.6 (4)	C10—C11—C12—C13	−174.2 (2)
F3A—C20A—C7—C8	49 (2)	C16—C11—C12—Cl1	−178.55 (18)
F1A—C20A—C7—C8	176.8 (13)	C10—C11—C12—Cl1	5.6 (3)
F2A—C20A—C7—C8	−65.8 (19)	C11—C12—C13—C14	−0.6 (4)
F3A—C20A—C7—C6	−130.1 (17)	Cl1—C12—C13—C14	179.5 (2)
F1A—C20A—C7—C6	−2.5 (18)	C12—C13—C14—C15	−1.1 (4)
F2A—C20A—C7—C6	114.9 (15)	C12—C13—C14—Cl2	178.5 (2)
F3A—C20A—C7—C20	−159 (16)	C13—C14—C15—C16	1.7 (4)
F1A—C20A—C7—C20	−31 (14)	Cl2—C14—C15—C16	−177.9 (2)
F2A—C20A—C7—C20	86 (15)	C14—C15—C16—C11	−0.7 (4)
F3—C20—C7—C8	4.5 (8)	C12—C11—C16—C15	−0.9 (4)
F2—C20—C7—C8	−124.1 (6)	C10—C11—C16—C15	174.7 (2)
F1—C20—C7—C8	123.6 (6)	C18—O1—C17—O2	−1.4 (4)
F3—C20—C7—C6	−176.7 (6)	C18—O1—C17—C2	178.7 (2)
F2—C20—C7—C6	54.7 (8)	C1—C2—C17—O2	9.3 (4)
F1—C20—C7—C6	−57.6 (8)	C3—C2—C17—O2	−171.8 (3)
F3—C20—C7—C20A	−24 (14)	C1—C2—C17—O1	−170.9 (2)
F2—C20—C7—C20A	−153 (15)	C3—C2—C17—O1	8.1 (4)
F1—C20—C7—C20A	95 (15)	C17—O1—C18—C19	176.4 (3)
C6—C7—C8—C9	−1.3 (4)		

### Hydrogen-bond geometry (Å, °)

**Table:**

*D*—H···*A*	*D*—H	H···*A*	*D*···*A*	*D*—H···*A*
C16—H16A···O3^i^	0.93	2.52	3.292 (5)	141
C18—H18A···F2^i^	0.97	2.50	3.355 (7)	147

Symmetry codes: (i) −*x*+1, −*y*+1, −*z*.
